# The effects of a peer-led training program on female students’ self-esteem in public secondary schools in Shiraz

**Published:** 2014-04

**Authors:** MOHAMMAD HOSSEIN KAVEH, MARYAM HESAMPOUR, LEILA GHAHREMANI, HAMID REZA TABATABAEE

**Affiliations:** 1 Department of Health Education and Promotion, Faculty of Health, Research Center for Health Sciences, Shiraz University of Medical Sciences, Shiraz, Iran;; 2 Department of Health Education and Promotion, Faculty of Health, Shiraz University of Medical Sciences, Shiraz, Iran;; 3 Department of Epidemiology, Faculty of Health, Shiraz University of Medical Sciences, Shiraz, Iran

**Keywords:** Self esteem, Adolescents, Education

## Abstract

**Introduction**: Low self-esteem in adolescence is one of the risk factors for negative outcomes in important domains of adulthood life. Due to the lack of trials based on modern methods of teaching in the field of self-esteem, this study aimed to investigate the effects of a peer-led training program on female second graders’ self-esteem in public secondary schools in Shiraz.

**Methods**: The present study is an educational controlled trial. 223 public school female students in the second grade were selected with the Multi-stage random cluster sampling method. The selected Schools were assigned randomly to experimental and control groups. The data were collected before, one and six weeks after an intervention in the control and experimental group, using Pope's 5-scale test of self-esteem with Cronbach's alpha reliability of 0.85. The educational intervention in the experimental group was a peer-led approach, using discussion techniques in small groups (the group work, role play and group play) and a 5-volume training manual. The data were analyzed through SPSS, version 14, using Mann-Whitney test, Chi-square test, Wilcoxon and repeated measure Analysis of Variance (ANOVA).

**Results**: The results showed that the mean of total self-esteem scores and the sub-scales (except for family self-esteem) in the experimental groups compared to that in the control groups, one and six weeks after the peer-led based approach intervention was significantly different (p<0.001). Before the intervention, the mean for self-esteem in the experimental groups was 51.80±13.91 but in the first post-test and second post-test the mean increased to 73.72±12.94, and 69.48±12.63, respectively. Before the educational intervention, the frequency distribution of females’ self-esteem in the experimental and control groups did not differ significantly from each other (p=0.337). But during one and six weeks after the intervention, a significant increase was observed between the two groups (p<0.001).

**Conclusion**: The results of this study suggest that peer education is an effective way to promote self-esteem in adolescents. Providing opportunities such as a peer-led approach can help adolescents to acquire practical ways to increase their self-esteem.

## Introduction

Self-esteem is one of the fundamental elements of character and important determinant of balance and self-transcendence (1). It is also an essential component of good health (2). Self-esteem is a “set of attitudes and beliefs that one has about self and the surrounding world” (3). From Pope’s viewpoint, “everyone’s self-esteem based on a combination of objective information about each person's and subjective values, ​​for which information is allowed, will be built” (4).

Adolescence is a critical period of physical, psychological, emotional, behavioral and cognitive change which are the determinants of health in adulthood, so protection of adolescents in this period appears vital and essential (5).

Low self-esteem is the most critical problem that adolescents face today in the field of mental health (6).

According to the existing evidence, low self-esteem in adolescents is related to negative consequences such as antisocial behavior and delinquency (6), academic failure (7), aggression (8), and suicide (9). In contrast, high self-esteem in adolescents is associated with happiness, self-confidence (10), academic achievement (11), community involvement, interpersonal relationships and mental health (3).

Self-esteem is the cognitive schema and acquisitive and learning phenomenon (2, 12). Social institutions such as family, school, peers and the media have an important role in the development of self-esteem (13).

School, as the first official status of social experience of adolescents, has an important role in the process of social development and is a decent environment for training programs in the field of self-esteem (7, 14). School-based programs, using respondent-driven and interactive techniques, have an effective role in the improvident of self-esteem (15).

The peer-led education approach is an effective behavior change strategy, increasing the power of thought, creativity, participation (16) and self-esteem (17). Peers provide the major source of feedback related to self-esteem, mutual support and opportunities for rehearsal of tasks in preparation for adulthood (18).

Peers are a group of individuals who have similarities in terms of age, socioeconomic status and interests (19). Belonging and attachments of peers to each other provide a rich environment for self-esteem development. Global self-esteem is influenced by cumulative experience that peers have had in the past (20).

Peer-led approach is a “process whereby well trained and motivated individuals undertake formal or informal activities with their peers” (21). The purpose of this approach is formation of knowledge, attitude, beliefs and skills of an individual so that he/she is empowered to be responsible for maintaining his/her own health (22).

Facilitation of the exchange of information and emotion, similar socioeconomic status of peer educator and learner, and having common interests (23) are features that increase the effectiveness of this approach.

Few studies have been conducted to examine the efficacy of this approach. The positive effects of this approach have been proved in females’ mental health (24), AIDS (21, 22), prevention of smoking (25), and breast self-examination (17). But a training trial based on peer-led approach to promote self-esteem was not found.

Therefore, with regard to research and knowledge gaps, it appears the trial of peer-led education program is useful for development of scientific knowledge in the field of promoting self-esteem in adolescents. This study is a dynamic step in this direction, aiming to investigate the effect of peer education program on self-esteem of female second graders in public secondary schools in Shiraz. Our assumptions in this study indicate level of self esteem and its subscales (general, family, academic, social, body) in students of experimental group after educational intervention is different in comparison with students in control group.

## Methods

This study is an educational randomized controlled trial. The sample consisted of 223 female second graders in secondary schools in Shiraz. A multi-stage cluster random sampling method was used. In the first stage, 4 educational districts of Shiraz, due to the similarity of socio-economic status, were considered in 2 categories (zones 1 and 2 in the first category, and zones 3 and 4 in the second category). Then, one area was selected from each category based on simple random sampling. Next, from each district, two schools were selected using the simple random sampling method. The selected schools in each district were randomly allocated to the experimental and control groups. In each school, two classrooms from the second grade were selected randomly. Finally, 115 students were in the experimental group and 108 in the control group; in total, 223 students participated in the study. 

Self esteem was evaluated using Pope's 5-Scale Test of self esteem for students. The test consists of 60 questions and evaluates self esteem on five scales: general, academic, family, social, and body scales. In pope's test, a lie scale was established to evaluate response validity (4).

Rating scale consists of responses to questions by always, sometimes or never and is scored 2, 1, 0 respectively; the negative items on the questionnaire were graded in the reverse order (4). The reliability coefficient of Emami and Fatehizadeh’s study was 0.85 (26); in Mazaheri’s study it was 0.73 and in the present study 0.80 has been reported (27).

In this study, self-esteem scores were categorized in four different groups. Students with self-esteem scores of below 40 were in the category 'weak'; those whose self-esteem scores were between 40 and 60 were in class, "middle", those with scores between 60 to 80 in the category "good" and finally, those whose self esteem scores were more than 80 were in the "excellent" category.

To evaluate the effectiveness of peer-led training on the students’ learning in the experimental group, a self-assessment questionnaire was used after training. The researcher-made questionnaire consisted of 14 key issues of educational content (e.g. my information on ways to boost my self-esteem before and after attending the training).             

The respondents evaluated and reported their level of knowledge in each subject before and after training on a 3-level grading scale (low, middle, high). For each item, for the highest level, the score was 3 and 1 for the lowest level. Scores ranged from 14 to 42. 

The experimental groups’ satisfaction with the learning process was measured using 13 questions [For example, “How much is the peer leading approach able to direct and control the class?”], with the 3 point Likert-scale (low, middle, high). Based on the students’ responses, learning and satisfaction, three levels were determined. Those with scores of less than 20.1 were at the undesirable level, between 20.1 to 32 were at desirable levels, and between 32.1 to 39 were in most desirable.

For comparison of the mean change in total score and subscales of self-esteem in both the experimental and control groups, the educational program was run in two parts. In the first part of the educational intervention, from each class, 6 volunteer students were selected based on the recommendation of the teacher and school administrator as peer educators. A total of 24 peer educators were selected to attend the intervention.

Then a two-day workshop was held for peer educators using discussion techniques in small groups (of teamwork, role playing and group games). In addition, 5-volume training manual and pamphlets (containing key point) were presented to them. It is noteworthy that training content provided by the researcher was matched for peer educators and students. 

In the second part, the peer educators were asked to teach their students the provided training content in a workshop using interactive techniques during 5 sessions of 90 minutes. In addition, the 5-volume training manual and pamphlets (containing key points) were also available to the students. The training topics discussed in the sessions included self-esteem and ways of fostering it, effective communication skills, problem solving and assertiveness skills.

In this regard, for implementation of learning activities in small groups, the students were organized in groups of 5-6. 

The data were collected using demographic information questionnaire and Pope Self-esteem before, one and six weeks after the educational intervention in the control and experimental groups. The collected data were, then, analyzed through SPSS statistical software, version 14 (SPSS Inc, Chicago, IL, USA). The statistical tests used were Chi-square, repeated measure Analysis of Variance (ANOVA) analysis and LSD multiple comparison tests. 

## Results

The statistical analysis showed no significant difference between the experimental and control groups concerning the students’ demographic variables. The students’ mean age in the experimental and control groups was 12.77±0.56 and 12.85±0.66, respectively. 32.2% of the students in the experimental and 27.8% of the students in the control groups were the second child of the family.

In the present study, the fathers of 37.4% of the students in the experimental and the fathers of 38% of those in the control group had university education, and their mothers had secondary school education and diploma [experimental (44.3%), and controls (42.6%)]. 

Also, the fathers of 9.6% of the students in the experimental and the fathers of 11.1% of those in the control group had elementary education. Their mothers had the same level of education [the experimental group (11.3%) and the control group (15.7%)].

A higher percentage of fathers in the experimental (41.7%) and control groups (38%) were self employed and a smaller percentage of them in the experimental (2.6%) and control (4.6%) groups were unemployed, and the rest were employed as, employees, workers or were retired. Most mothers in the experimental (77.4%) and control groups (77.8%) were housewives and the rest were employed [experimental (22.2%) and control groups (22.2%)].

The mean scores of total self-esteem in the experimental and control groups were 51.80±13.91 and 53.57±14.76 respectively (Table 1).

The mean scores of total self-esteem in the experimental group increased to 73.72±12.94 in the first post-test (Table 1).

Although the mean±SD of total self-esteem in the experimental group decreased a little after the second post-test (69.48±12.63), it was still higher than the pre-test level. The mean scores of total self-esteem in the control group in the first and second post-tests were 49.69±14.72 and 46.37±17.21 respectively (Table 1).

Using repeated measurement analysis and LSD multiple comparison test, we found that the changes through phases of measurements in total self-esteem mean scores and in the subscales (except for family self-esteem) within and between the study groups were statistically significant (p<0.001) (Table 1).

Using repeated measurement test and LSD, we found that the family self-esteem subscale was significantly higher in the experimental group (p<0.001).

In the present study, after the educational intervention in the experimental group, the general self-esteem and academic self-esteem showed the greatest change and family self-esteem had the lowest change during the study. In the control group, self-esteem and its sub-scales declined. After the intervention, the highest self-esteem in the control group students was that of family and highest decline was in general and social self-esteems (Table 1).

**Table 1:** Comparison of the mean scores of self esteem between the study groups through phases of measurement

**Groups** **Self esteem and subscale**		**Pre-program**	**One week after program**	**6 week after program**	**p (within groups)**
**General scale**	**Experimental**	9.59±3.21	15.74±3.07	15.12±3.19	p<0.001
	**Control**	10.39±4.11	9.37±4.09	8.40±4.36	p<0.001
**p (between groups)**	p<0.001				
**Academic scale**	**Experimental**	8.98±4.06	14.33±3.31	13.05±3.26	p<0.001
	**Control**	9.63±3.36	9.17±3.43	8.85±3.95	p=0.004
**p (between groups)**	p<0.001				
**Social scale**	**Experimental**	10.61±3.53	14.67±3.22	14.03±3.05	p<0.001
	**Control**	10.31±3.03	9.18±3.25	8.31±3.73	p<0.001
**p (between groups)**	p<0.001				
**Family scale**	**Experimental**	12.43±4.50	13.05±4.12	12.70±3.57	p=0.529
	**Control**	12.13±3.42	11.37±3.65	10.99±4.19	p<0.001
**p (between groups)**	p<0.001				
**Baby scale**	**Experimental**	10.18±4.04	15.93±2.83	14.57±3.24	p<0.001
	**Control**	11.12±4.37	10.60±4.32	9.81±4.93	p<0.001
**p (between groups)**	p<0.001				
**Total scale**	**Experimental**	51.80±13.91	73.72±12.94	69.48±12.63	p<0.001
	**Control**	53.57±14.76	49.69±14.72	46.37±17.21	p<0.001
**p (between groups)**	p<0.001				

As shown in Table 2, before the educational intervention, the frequency distribution in the experimental and control groups in terms of total self-esteem levels did not differ significantly from each other (p=0.337). But during a week and six weeks after the intervention, a significant difference was observed between the two groups (p<0.001). Before the educational intervention, 20.9% of the experimental group students were in the weak class. This decreased to 0.9% during a week and six weeks after the intervention.

**Table 2:** Comparison of the frequency distribution of levels of total self esteem between the study groups through phases of measurement

**Levels of self esteem**	**Pre-program**		**One week after program**		**6 week after program**	
	**Control** ** group**	**Experimental group**	**Control** ** group**	**Experimental group**	**Control** ** group**	**Experimental group**
	**Frequency(%)**	**Frequency(%)**	**Frequency(%)**	**Frequency(%)**	**Frequency(%)**	**Frequency(%)**
**Weak**	22(20.4)	24(20.9)	33(30.6)	1(0.9)	46(42.6)	1(0.9)
**Moderate**	54(50.0)	61(53.0)	51(47.2)	21(18.3)	39(36.1)	26(22.6)
**Good**	28(25.9)	27(23.5)	22(20.4)	50(43.5)	19(17.6)	59(51.3)
**Excellent**	4(3.7)	3(2.6)	2(1.9)	43(37.4)	4(3.7)	29(25.2)
**Total**	108(100.0)	115(100.0)	108(100.0)	115(100.0)	108(100.0)	115(100.0)
**Mann-Whitney test**	p=0.337		p<0.001		p<0.001	

The median score of knowledge in the experimental group was 23 before the intervention and after the intervention it increased to 39. Wilcoxon statistical test showed statistically significant differences in these students (p<0.001) (Table 3).

**Table 3:** Comparison of the mean scores of knowledge in students, before and after of intervention in experimental group

**Experimental group**					
**Knowledge student**	**Mean±SD**	**Med**	**Score range [13-65]**		**Wilcoxon** ** signed test** **p**
			**Lower**	**Upper**	
**Before program**	23.80±6.63	23	14	41	p<0.001
**After program**	39.89±3.58	39	24	42	

After the intervention, the mean and median scores of the teaching process evaluation and students’ satisfaction in the experimental group were 34.46±4.19 and 36. In assessing teaching process and students’ satisfaction, the range of scores was 39 to 13. In this evaluation, 78(67.8%) students reported the learning process very well, 36(27.8%) of them said that it was desirable, and 2 students (1.7%) assessed it less than the desirable level. The Chi-square test showed no significant difference between the levels of education process and students’ satisfaction (p<0.001) (Table 4).

**Table 4:** Comparison of the levels of satisfaction in experimental group, after intervention

**Experimental group (after intervention)**			
**Levels of satisfaction**	**Frequency(%)**	**Mean±SD**	**Chi-square test** **p**
**Undesirable**	2(1.7)	34.46±4.19	p<0.001
**Desirable**	35(30.4)		
**Most desirable**	78(67.8)		

## Discussion

This study showed that peer-led approach resulted in a significant increase in total self-esteem in female students' of the experimental group one and six weeks after the intervention. National and international studies have confirmed the efficacy of peer-led approach. This approach is an effective strategy for HIV prevention (21, 22, 28, 29), preventing smoking (25), breast self-examination training (17), and parenting in the context of children behavioral disorder (30).

Head in his results show that the peer led parenting intervention significantly reduced child behavior problems and improved parenting competencies (30). Also, Abu-Saeed in his study concluded that training by peers is an effective approach for HIV prevention in adolescents (28).

The results of a study aiming to compare the peer-led and teacher-led approach showed that peer-led programs are more popular for students (31).

In another study, it was shown that the training method with peer-led approach has improved the health of adolescent girls. Also, peer approach led to young people’s empowerment, provided opportunities for them to participate in activities and improving communication between mothers and daughters (16). Therefore, it can be concluded that in the present study, improving self-esteem in the students of experimental group was because of that the peer educators have model role for their peers.

In the present study, the students’ knowledge increased significantly after the intervention indicating that the implemented training program could meet the students’ needs considerably. Furthermore, the students’ high satisfaction with peer education program confirms the effectiveness of the program.

Bulduk, etal’s study showed that training through peer group would lead to increased students' knowledge in the field of physical diseases and HIV transmission (22).

The results of the present study are also in compliance jalali (10), Neacşu (12), Taylor (32), Waite (33), Borras (34), Kiani (35) and Yoo (14) findings concerning the impact of training on self-esteem. Acquisitive and learning of self-esteem is also supported by this study.

Also, the results of the present study showed that the self-esteem subsets (general, body, social and academic), except for the family self-esteem had a significant increase after the training intervention.

It is noteworthy that similar studies are very limited to compare the present study with the other similar studies in any one of the self-esteem sub-scales.

In present study, general self-esteem of the students showed the greatest change in the experimental group. These results consistent with daglas, Neacşu, Kermanshahi studies (6, 12, 15). Neacşu showed that cognitive-behavioral program improve total self-esteem and leads to a significant reduction in stress levels of the participants after training (12).

Kermanshahi in his study concluded that the use of educational group therapy program increase the self-esteem and improve the mental health of adolescent girls. Obviously, Adolescents tend to engage in group discussions with the peers. Group’s Members said that this method was an effective way to express their problems and reach solutions (6).

According to the findings of this study, self-esteem is influenced by body image component. In this study, the peer educators, due to their dominance over their peers, could make positive changes concerning the students’ body image and thus significantly increase their body self-esteems. 

Findings in other studies approved that low self-esteem in body dissatisfaction is important and necessary. Paxton and Clay et al in their studies concluded that educational intervention can help to rebuild bad image of female body formed (36, 37). Katibaei and et.al showed in their study that have a good body image and reinforcing physical abilities is one of the key elements of self-esteem in adolescents and training have a special role in forming this good image (38).

In the present study, participating in group counseling sessions and interaction with peers may also reinforce the belief that students learn using others’ opinion to solve the problem of herself/himself and thereby it increases the social self-esteem. 

Dalgas and et-al in their study about the effects of intervention programs on the self-esteem of school-age students showed that after the educational intervention, general self-esteem and social self-esteem of students have increased considerably (15).

In this study, after general self-esteem, academic self-esteem in the experimental group showed the greatest increase. The positive effect of education on promoting self-esteem, relaxation, behavioral changes and academic achievement has been proven in several studies (1, 2, 39).

In the present study, peer-led approach training intervention was undertaken in school. Peer group are more closes in terms of education and peer relationships in School. Peer led approach could have positive effects on students academic performance in terms of self-esteem in the school environment. Results of other studies have also confirmed this. Results of studies of Jalali (10), Taylor and Montgomery (32) and Hughes (40) approved the self-esteem training positive effects on academic achievement.

This study did not find a statistically significant increase in family self-esteem. Hui and Ryan’s studies show that students who do not have the warm support of families have lower self-esteem are more involved in anti-social behavior (41, 42). Here the role of parents is of great importance. Thus, in addition to students, their parents should be trained in promoting the student’s self-esteem to increase the students' family self-esteem.

The results of the present study showed that the total self-esteem and its subscales in the control group had a decreasing trend. This finding is consistent with those from previous studies. Studies showed that self-esteem is changing along life period, and in adolescence it declines due to the critical nature of this course (5, 43). Overall, the results of this study and the findings of other studies proved the higher self-esteem index in the experimental group than the control group after the intervention. These findings suggest a significant impact of peer-led approach on improving self-esteem.

## Conclusion

According to these results, designing training programs based on peer-led approach can be effective in improving the students' self-esteem. Providing opportunities such as peer-led approach which leads the adolescents to learn practical ways to increase self-esteem is necessary. Schools can have a significant role in this regard by providing training programs for both students and parents. 

**Limitations and suggestion**

In this study, peer-led approach influenced female students in the secondary schools; however, the effect of this approach has not yet been confirmed on males. Random selection of students was performed in only two of four districts of education. In this study, the data were collected in the form of self-report. Using this technique in subjects who were adolescents may be more or less associated with bias. However, in other studies, the same method was used to measure self-esteem. Given the above limitations, generalizing the results to the whole community should be done cautiously.

Due to the mentioned constraints, evaluating the impact of peer led approach on self-esteem in boys and girls at different educational levels is proposed. In addition, for comparing the impact of this approach on self-esteem in four districts of education should be examined. 

**Ethical consideration**

Prior to performing this research, approval of the director of education was obtained and after explanation about the study, the participants signed the written consent forms. 

Also participants in the study were assured that the questionnaire will be anonymous and all information will remain confidential. Upon completion of the study, in order to maintain the ethical considerations, we provided the control group with the key points of the educational program.

**Implications**

The findings of this research could help the educators involved in the education department or the counseling centers on adolescent health to increase self-esteem. The role of self-esteem and its positive role in the performance of students in the academic and social life confirm the significance of this.
